# Vitamin D insufficiency and cardiovascular involvement in systemic sclerosis: Association with echocardiographic parameters and risk factors

**DOI:** 10.1016/j.ijcrp.2025.200502

**Published:** 2025-08-26

**Authors:** Gianluca Pagnoni, Dilia Giuggioli, Marco de Pinto, Arianna Maini, Elisa Battigaglia, Pierluca Macripò, Amelia Spinella, Giulia Olivetti, Antonio Manenti, Marcello Pinti, Giuseppe Boriani, Daniela Aschieri, Anna Vittoria Mattioli, Francesco Fedele, Francesca Coppi

**Affiliations:** a Cardiology Unit of Emergency Department, Guglielmo da Saliceto Hospital, 29121, Piacenza, Italy; b Rheumatology Unit, Azienda Ospedaliero-Universitaria Policlinico di Modena, University of Modena and Reggio Emilia, Modena, Italy; c Department of Medical and Surgical Sciences for Children and Adults. University of Modena and Reggio Emilia, Via del Pozzo 71, 41124, Modena, Italy; d Cardiology Division, Department of Biomedical Metabolic and Neural Sciences, University of Modena and Reggio Emilia, Via del Pozzo 71, 41124, Modena, Italy; e Anesthesia and Intensive Care Medicine, Policlinico Di Modena, University of Modena and Reggio Emilia, Via del Pozzo 71, 41124, Modena, Italy; f Department of Surgical, Medical and Dental Department of Morphological Sciences related to Transplant, Oncology and Regenerative Medicine, University of Modena and Reggio Emilia, Modena, Italy; g National Institute for Cardiovascular Research (INRC), Via Irnerio 48, 40126, Bologna, Italy; h Department of Life Sciences, University of Modena and Reggio Emilia, Via G. Campi 287, 41125, Modena, Italy; i Department of Quality of Life University of Bologna - Alma Mater Studiorum, Bologna, Italy; j Emeritus Professor of Cardiology, Sapienza University of Rome, Italy; k National Institute for Cardiovascular Research (INRC), Bologna, Italy

**Keywords:** Vitamin D insufficiency, Systemic sclerosis, Cardiovascular diseases, Echocardiography, Risk factors, Pulmonary hypertension

## Abstract

**Background:**

Vitamin D plays several roles beyond bone metabolism, potentially protecting the cardiovascular system. Systemic sclerosis (SSc) is an autoimmune disease characterized by fibrosis and vascular dysfunction, carrying a high cardiovascular risk. However, the relationship between vitamin D insufficiency and cardiovascular involvement in SSc patients remains unclear. This study aims to assess the association between low vitamin D levels, echocardiographic parameters indicative of ventricular dysfunction, and cardiovascular risk factors in SSc patients.

**Methods:**

A retrospective observational study involved 160 SSc patients undergoing echocardiography and serum 25-hydroxyvitamin D measurement. Patients were categorized into two groups: vitamin D insufficiency (<30 ng/mL) and normal levels (≥30 ng/mL). Echocardiographic parameters and cardiovascular risk factors were evaluated.

**Results:**

Vitamin D insufficiency was detected in 68.9 % of patients. Patients with insufficient vitamin D had higher systolic pulmonary arterial pressure (PAPs; 37.68 ± 7.56 mmHg vs. 33.12 ± 6.17 mmHg; p = 0.004), reduced TAPSE/PAPs ratio (0.65 ± 0.13 vs. 0.72 ± 0.14; p = 0.021), increased interventricular septal thickness (8.17 ± 1.28 mm vs. 7.69 ± 1.18 mm; p = 0.028), and greater left ventricular end-diastolic diameter (44.06 ± 4.28 mm vs. 42.67 ± 3.44 mm; p = 0.037), indicating ventricular dysfunction. Vitamin D insufficiency significantly correlated with hypertension (OR = 2.31; p = 0.032), dyslipidemia (OR = 2.45; p = 0.015), and overweight/obesity (OR = 4.73; p = 0.002), but not with diabetes or smoking.

**Conclusions:**

Vitamin D insufficiency may contribute to cardiovascular dysfunction in SSc patients. Monitoring vitamin D levels might improve cardiovascular risk stratification. Further studies are necessary to determine if supplementation could enhance cardiac outcomes.

## 1 Introduction

Vitamin D, beyond its well-established role in bone metabolism, has gained increasing scientific attention for its potential beneficial effects on the cardiovascular system [1,2]

Numerous studies suggest that vitamin D may positively influence various cardiovascular parameters, reducing the risk of hypertension, atherosclerosis, and other cardiac diseases through complex mechanisms, including modulation of the renin-angiotensin-aldosterone system (RAAS), endothelial function regulation, and attenuation of inflammation [3,4].

This effect is particularly relevant in hypertensive patients, where RAAS hyperactivation often underlies disease progression, potentially reducing the risk of left ventricular hypertrophy and heart failure. Additionally, vitamin D acts directly on vascular smooth muscle and endothelial cells, enhancing endothelial function through increased nitric oxide (NO) production, thus promoting vasodilation and reducing vascular inflammation, preventing atherosclerosis [5,6].

Vitamin D also decreases oxidative stress in endothelial cells, modulating endothelial nitric oxide synthase (eNOS) activity, counteracting NO degradation by reactive oxygen species (ROS), inhibiting NADPH oxidase, and enhancing cellular antioxidant capacity [7].

Another cardioprotective mechanism of vitamin D involves regulation of calcium metabolism in cardiac cells, crucial for myocardial contractility and arrhythmia prevention [6].Studies in murine cardiomyocytes have demonstrated both genomic and non-genomic effects, highlighting the critical role of the vitamin D receptor (VDR), particularly localized in cardiac T-tubules, in maintaining myocardial function [8].

Furthermore, vitamin D exerts immunomodulatory effects that indirectly influence cardiovascular health by decreasing pro-inflammatory cytokines, such as tumor necrosis factor-alpha (TNF-α) and interleukin-6 (IL-6), which are associated with increased cardiovascular risk, including myocardial infarction and stroke [7,9].

While epidemiological studies have often shown an inverse association between serum vitamin D levels and cardiovascular disease incidence, interventional trials have yielded conflicting results. Some suggest benefits limited mainly to patients with pre-existing deficiency [10,11]. In contrast, the VITAL study, involving over 25,000 healthy adults followed for 5.3 years, found no significant reduction in cardiovascular events with daily vitamin D_3_ supplementation (2000 IU) versus placebo [12].

Similarly, a systematic review and meta-analysis of 11 randomized controlled trials in hypertensive patients showed only a modest reduction in diastolic blood pressure and no significant effect on systolic blood pressure, with no notable benefit in normotensive individuals [13].

In summary, although biological plausibility and epidemiological evidence support a protective cardiovascular role for vitamin D, supplementation benefits appear inconsistent, underscoring the need for further targeted research.Systemic sclerosis (SSc) is a chronic autoimmune disease characterized by tissue fibrosis, vasculopathy, and autoimmunity, involving excessive extracellular matrix deposition, autoantibody production, and structural organ damage [14,15].

Cardiovascular involvement in systemic sclerosis represents one of the major determinants of morbidity and mortality. Patients with SSc exhibit an increased risk for pulmonary hypertension, myocardial fibrosis, conduction system abnormalities, and left ventricular diastolic dysfunction, all of which significantly worsen prognosis and quality of life. Pulmonary hypertension, with a prevalence of up to 12 % in SSc, is particularly impactful on survival. Myocardial fibrosis, detectable by advanced imaging, is linked to arrhythmias, impaired ventricular function, and poor outcomes [[16], [17], [18], [19], [20]].

Beyond cardiovascular complications, gastrointestinal involvement in SSc may contribute to vitamin D deficiency through malabsorption mechanisms such as small bowel disease, bacterial overgrowth, and exocrine pancreatic insufficiency, all of which impair fat-soluble vitamin absorption [[21], [22], [23]].

This study aims to evaluate the impact of vitamin D insufficiency on the cardiovascular system in patients with SSc. Specifically, it seeks to analyze the association between low vitamin D levels and both structural and functional echocardiographic alterations, with a particular focus on parameters indicative of right and left ventricular dysfunction.

Additionally, the study investigates the relationship between vitamin D insufficiency and major cardiovascular risk factors, including hypertension, dyslipidemia, obesity, and smoking habits, to determine whether vitamin D may modulate the inflammatory, metabolic, and vascular processes involved in the cardiovascular pathogenesis of these patients.

The ultimate objective is to assess whether vitamin D insufficiency may represent an independent risk factor for cardiac involvement in SSc patients and whether its monitoring could contribute to improved cardiovascular risk stratification in this population.

## 2 Materials and methods

### 2.1 Patients and study design

This retrospective observational study was designed to assess the prevalence of vitamin D (Vit D) insufficiency in patients with SSc and its association with echocardiographic alterations.

The study was approved by the local ethics committee of Area Vasta Emilia Nord (protocol no. 275/16), and informed consent was obtained from all participants before enrollment in the study. The study was conducted in accordance with Good Clinical Practice Guidelines and the World Medical Association Declaration of Helsinki [24].

A total of 160 consecutive patients with a confirmed diagnosis of SSc were enrolled. The diagnosis of systemic sclerosis (SSc) was established according to the American College of Rheumatology (ACR)/European League Against Rheumatism (EULAR) 2013 classification criteria, which include skin thickening of the fingers extending proximal to the metacarpophalangeal joints (sufficient criterion), and additional criteria such as fingertip lesions, telangiectasia, abnormal nailfold capillaries, Raynaud's phenomenon, and SSc-related autoantibodies [[25], [26], [27]].

These patients were referred to the Cardiology Unit of the Policlinico di Modena for screening echocardiography.

All patients were enrolled as part of a structured cardiopulmonary screening program aimed at the early detection of pulmonary hypertension and other cardiovascular complications in systemic sclerosis, conducted between March 2022 and July 2024.

Inclusion criteria comprised patients aged 18 years or older with a confirmed diagnosis of SSc. Exclusion criteria included a history of cardiovascular diseases unrelated to SSc, multiple autoimmune conditions, active systemic infections, or malignancies.

Baseline demographic and clinical data were systematically collected at enrollment, including age, sex, BMI, eGFR (CKD-EPI), disease duration from the first non-Raynaud symptom, office blood pressure (mean of three consecutive measurements in a seated position), and circulating levels of heart failure biomarkers (BNP).

Medical history was collected through a structured interview to identify major cardiovascular risk factors (CV RF), including smoking status (current or past), body mass index (BMI) with overweight/obesity classified as BMI >25 kg/m^2^, hypertension was defined as office blood pressure ≥140/90 mmHg (mean of three consecutive seated measurements) or current antihypertensive therapy, dyslipidemia identified by ongoing lipid-lowering therapy or LDL levels >115 mg/dL, and a diagnosis of type 1 or type 2 diabetes mellitus.

In addition to the assessment of major cardiovascular risk factors, information was collected on renal function, use of antihypertensive and lipid-lowering therapies, and the presence of other ongoing cardiovascular treatments. For each patient, the latitude of residence and the season in which vitamin D measurement was performed were recorded to account for potential effects of geographic location and seasonal variation on vitamin D status. Data on dietary calcium and phosphorus intake, as well as direct measures of central adiposity (e.g., waist circumference or waist-to-hip ratio), were not available, representing a limitation of the present study.

Sample size calculation was based on previous literature reporting a high prevalence of vitamin D insufficiency in systemic sclerosis patients, ranging between 60 % and 80 % [28,29]. Assuming an expected prevalence of approximately 70 %, with an acceptable margin of error of ±7 % and a confidence interval of 95 %, a minimum sample size of approximately 160 patients was estimated using standard statistical formulas for prevalence studies.

### 2.2 Echocardiographic assessment and laboratory analysis

Echocardiographic assessment was performed using a Philips EPIQ ultrasound system by two experienced cardiologists with specific expertise in echocardiography. All echocardiographic measurements and interpretations were standardized according to guidelines provided by the American Society of Echocardiography and the European Association of Cardiovascular Imaging. Structural and functional parameters of both right and left heart chambers were evaluated [30].

Serum 25-hydroxyvitamin D levels were measured at the Clinical Laboratory using an enzyme-linked immunosorbent assay (ELISA), specifically designed for vitamin D quantification. Vitamin D insufficiency was defined as serum levels below 30 ng/mL, based on widely accepted clinical criteria. Levels above this threshold were considered sufficient [5,28,31,32].

### 2.3 Statistical analysis

Statistical analysis was performed using SPSS software version 30.0.0, with statistical significance set at p < 0.05. The distribution of variables was assessed using the Shapiro-Wilk test. For group comparisons, continuous variables were analyzed using the Student's t-test for independent samples or the Mann-Whitney *U* test for non-normally distributed variables. Categorical variables were compared using the chi-square (χ^2^) test or Fisher's exact test when expected frequencies were below 5 in any cell. Pearson's correlation coefficient was used to explore relationships between vitamin D levels, echocardiographic parameters, and cardiovascular risk factors. Data were presented as mean ± standard deviation (SD) for normally distributed variables, median and interquartile range (IQR) for non-normally distributed variables, and percentages for categorical variables.

## 3 Results

The study included a total of 160 consecutively recruited patients with systemic sclerosis. The sex distribution revealed a marked predominance of women, with 140 female participants (87.5 %) and 20 male participants (12.5 %), reflecting the well-known gender prevalence of SSc.

The mean age of the study population was 60.5 ± 12.7 years, ranging from 22 to 86 years. Age distribution was further described through quartiles: the first quartile corresponded to 52 years, the median to 61 years, and the third quartile to 71 years, indicating a population mainly composed of late-adulthood individuals.

The median disease duration from the first non-Raynaud symptom was 8 years (IQR 4–14).

Mean systolic and diastolic blood pressures were 126 ± 18 mmHg and 77 ± 11 mmHg, respectively. Median BNP was 45 pg/mL (IQR 28–73).

The mean body mass index (BMI) was 23.4 ± 4.1 kg/m^2^, with a minimum value of 15.0 kg/m^2^ and a maximum of 39.0 kg/m^2^. The median BMI was 23.0 kg/m^2^. Additionally, 23.8 % of the population (38 patients) had a BMI greater than 25 kg/m^2^, classifying them as overweight or obese according to WHO criteria. The mean body surface area (BSA) was 1.67 ± 0.16 m^2^, with values ranging from 1.30 m^2^ to 2.45 m^2^. The median BSA was 1.66 m^2^, with a first quartile of 1.56 m^2^ and a third quartile of 1.78 m^2^.

Renal function was within normal range in most patients, with a mean serum creatinine of 0.86 ± 0.21 mg/dL (minimum 0.52 mg/dL; maximum 1.38 mg/dL) and a mean estimated glomerular filtration rate (eGFR) of 88.3 ± 14.6 mL/min/1.73 m^2^ (CKD-EPI), with values ranging from 54 to 126 mL/min/1.73 m^2^. A total of 7.5 % of patients had an eGFR <60 mL/min/1.73 m^2^. Antihypertensive therapy was used by 35.6 % of patients, most commonly angiotensin-converting enzyme inhibitors (ACE inhibitors) [enalapril, lisinopril] in 14.4 %, angiotensin II receptor blockers (ARBs) [losartan, valsartan] in 10.6 %, calcium channel blockers [amlodipine, nifedipine] in 6.9 %, and beta-blockers [bisoprolol, metoprolol] in 3.7 %. Lipid-lowering agents were prescribed to 31.2 % of patients, primarily statins [atorvastatin, rosuvastatin] in 27.5 % and ezetimibe in 3.7 %. Regarding environmental factors, all patients resided between 44° and 46° N latitude. Vitamin D measurements were distributed across the year as follows: spring 26.3 %, summer 21.9 %, autumn 28.8 %, and winter 23.0 %.Table 1.

**Table 1:** Baseline demographic, anthropometric, renal, cardiovascular, and environmental characteristics of the study population. Data are expressed as mean ± standard deviation (SD), median and interquartile range (IQR), or number (percentage), as appropriate. eGFR = estimated glomerular filtration rate; BP = blood pressure; BNP = brain natriuretic peptide.

Variable	Value
**Demographics**	
Female sex, n (%)	140 (87.5)
Age, years, mean ± SD (range)	60.5 ± 12.7 (22–86)
Disease duration, years, median (IQR)	8 (4–14)
**Anthropometric measures**	
Body mass index (BMI), kg/m^2^, mean ± SD (range)	23.4 ± 4.1 (15.0–39.0)
BMI >25 kg/m^2^, n (%)	38 (23.8)
Body surface area (BSA), m^2^, mean ± SD (range)	1.67 ± 0.16 (1.30–2.45)
**Renal function**	
Serum creatinine, mg/dL, mean ± SD (range)	0.86 ± 0.21 (0.52–1.38)
eGFR, mL/min/1.73 m^2^, mean ± SD (range)	88.3 ± 14.6 (54–126)
eGFR <60 mL/min/1.73 m^2^, n (%)	12 (7.5)
**Blood pressure**	
Systolic BP, mmHg, mean ± SD	126 ± 18
Diastolic BP, mmHg, mean ± SD	77 ± 11
**Cardiac biomarkers**	
BNP, pg/mL, median (IQR)	45 (28–73)
**Comorbidities and therapies**	
Hypertension, n (%)	57 (35.6)
Dyslipidemia, n (%)	110 (68.75)
Diabetes mellitus, n (%)	10 (6.3)
Current or past smoking, n (%)	67 (41.9)
Antihypertensive therapy, n (%)	57 (35.6)
Lipid-lowering therapy, n (%)	50 (31.2)
**Environmental factors**	
Latitude of residence, °N	44–46
Season of vitamin D measurement, n (%)	Spring 42 (26.3), Summer 35 (21.9), Autumn 46 (28.8), Winter 37 (23.0)

**Table 2:** The table presents the mean values (± standard deviation) of key echocardiographic parameters, comparing patients with normal vitamin D levels (≥30 ng/mL) and those with reduced levels (<30 ng/mL). DTD: left ventricular end-diastolic diameter; IVS: interventricular septal thickness; PW: left ventricular posterior wall thickness; Aortic sinus: aortic sinus diameter; Ascending aorta: ascending aorta diameter; Indexed LV mass: left ventricular mass indexed to body surface area; Wall thickness: left ventricular wall thickness; Indexed LA volume: left atrial volume indexed to body surface area; Indexed EDV: indexed left ventricular end-diastolic volume; Indexed ESV: indexed left ventricular end-systolic volume; EF: left ventricular ejection fraction; TAPSE: tricuspid annular plane systolic excursion; PAPs: systolic pulmonary arterial pressure; TRV: tricuspid regurgitation velocity; S': right ventricular longitudinal systolic velocity; TAPSE/PAPs: TAPSE to PAPs ratio, indicative of right ventricular function; RA area: right atrial area. p-values indicate statistically significant differences between groups, with p < 0.05 considered significant.

No.	Variable	Mean ± SD (VIT D Normal)	Mean ± SD (VIT D Reduced)	p-value
1	DTD (mm)	42.67 ± 3.44	44.06 ± 4.28	**0.037**
2	IVS (mm)	7.69 ± 1.18	8.17 ± 1.28	**0.028**
3	PW (mm)	7.76 ± 1.25	8.07 ± 1.26	0.166
4	Aortic sinus (mm)	28.47 ± 3.53	29.48 ± 3.99	0.123
5	Ascending aorta (mm)	28.62 ± 3.59	29.75 ± 4.27	0.100
6	Indexed LV mass (g/m^2^)	94.58 ± 16.23	98.34 ± 17.90	0.083
7	Wall thickness (mm)	9.13 ± 1.23	9.47 ± 1.36	0.104
8	Indexed LA volume (mL/m^2^)	26.34 ± 5.78	27.42 ± 6.15	0.148
9	Indexed EDV (mL/m^2^)	68.15 ± 10.42	70.36 ± 11.24	0.190
10	Indexed ESV (mL/m^2^)	25.31 ± 5.03	26.12 ± 5.21	0.232
11	EF (%)	55.21 ± 5.32	54.78 ± 5.50	0.583
12	TAPSE (mm)	22.47 ± 3.82	21.88 ± 4.05	0.345
13	PAPs (mmHg)	33.12 ± 6.17	37.68 ± 7.56	**0.004**
14	TRV (m/s)	2.78 ± 0.41	2.85 ± 0.44	0.315
15	S' (cm/s)	10.56 ± 2.05	10.32 ± 2.14	0.495
16	TAPSE/PAPs	0.72 ± 0.14	0.65 ± 0.13	**0.021**
17	RA area (cm^2^)	14.12 ± 3.26	14.47 ± 3.45	0.487

Overall, the examined population was characterized by a predominance of female subjects, an average age within the late-adulthood range, and a prevalence of BMI and BSA values typical of a population that is not severely overweight or obese.

**Table 3:** Reports the percentage distribution of key binary echocardiographic variables in patients with normal vitamin D levels (≥30 ng/mL) and those with reduced levels (<30 ng/mL). Pericardial effusion: presence of pericardial effusion; Concentric remodeling: concentric remodeling of the left ventricle; Concentric hypertrophy: concentric left ventricular hypertrophy; Eccentric hypertrophy: eccentric left ventricular hypertrophy; Diastolic dysfunction: overall diastolic dysfunction; Diastolic dysf. I/III: grade I diastolic dysfunction; Diastolic dysf. II/III: grade II diastolic dysfunction; Diastolic dysf. III/III: grade III diastolic dysfunction. Values are expressed as percentages with confidence intervals (CI), and the p-value indicates the level of statistical significance.

No.	Variable	Percentage (VIT D Normal)	Percentage (VIT D Reduced)	p-value
1	Pericardial effusion	8.33 % (CI: 3.29–19.55)	9.73 % (CI: 5.52–16.59)	1.000
2	Concentric remodeling	16.67 % (CI: 8.70–29.58)	18.58 % (CI: 12.49–26.75)	0.826
3	Concentric hypertrophy	2.08 % (CI: 0.37–10.90)	3.54 % (CI: 1.39–8.75)	1.000
4	Eccentric hypertrophy	0.00 % (CI: 0.00–7.41)	1.77 % (CI: 0.49–6.22)	1.000
5	Diastolic dysfunction	33.33 % (CI: 21.68–47.46)	40.71 % (CI: 32.10–49.93)	0.479
6	Diastolic dysf. I/III	29.17 % (CI: 18.29–43.30)	33.63 % (CI: 25.73–42.68)	0.663
7	Diastolic dysf. II/III	4.17 % (CI: 1.14–14.06)	7.08 % (CI: 3.59–13.53)	0.694
8	Diastolic dysf. III/III	2.08 % (CI: 0.37–10.90)	2.65 % (CI: 0.99–6.91)	1.000

**Table 4:** reports the correlation between vitamin D insufficiency (<30 ng/mL) and major cardiovascular risk factors, renal function, pharmacological therapies, and seasonal distribution of blood sampling. Data are expressed through Pearson's correlation coefficient, odds ratio (OR), and p-value. Statistically significant associations were found with hypertension (OR = 2.31, p = 0.032), dyslipidemia (OR = 2.45, p = 0.015), overweight/obesity (BMI >25 kg/m^2^) (OR = 4.73, p = 0.002), antihypertensive therapy (OR = 2.12, p = 0.041), and lipid-lowering therapy (OR = 1.88, p = 0.045). No significant correlation was observed with smoking, diabetes mellitus, reduced renal function (eGFR <60 mL/min/1.73 m^2^), or winter season blood sampling (p > 0.05).

Variable	Coeff. Pearson	Odds ratio	P-value
Reduced vitamin D → Hypertension	0.17	**2.31**	**0.032**
Reduced vitamin D → Smoking/Ex-Smoking	0.165	/	0.06
Reduced vitamin D → Dyslipidemia	0.18	**2.45**	**0.015**
Reduced vitamin D → Diabetes Mellitus	0.11	/	0.28
Reduced vitamin D → BMI >25	0.234	**4.73**	**0.002**
Reduced vitamin D → eGFR <60 mL/min/1.73 m^2^	0.09	/	0.21
Reduced vitamin D → Antihypertensive therapy	0.16	**2.12**	**0.041**
Reduced vitamin D → Lipid-lowering therapy	0.14	**1.88**	**0.045**
Reduced vitamin D → Winter blood sampling	0.12	/	0.08

A total of 110 out of 160 patients (68.9 %) had vitamin D levels below 30 ng/mL, indicative of insufficiency.

The analysis of continuous echocardiographic variables revealed statistically significant differences between the group with normal vitamin D levels and the group with reduced levels (<30 ng/mL). Specifically, patients with reduced vitamin D levels had a significantly larger mean left ventricular end-diastolic diameter (LVEDD: 44.06 ± 4.28 mm vs. 42.67 ± 3.44 mm; p = 0.037), suggestive of early ventricular remodeling. The mean interventricular septal thickness (IVS) was also significantly greater in the group with reduced vitamin D (8.17 ± 1.28 mm vs. 7.69 ± 1.18 mm; p = 0.028), potentially reflecting subclinical myocardial fibrosis or hypertrophy. Systolic pulmonary arterial pressure (PAPs) was significantly higher in patients with vitamin D insufficiency (37.68 ± 7.56 mmHg) compared to those with sufficient levels (33.12 ± 6.17 mmHg; p = 0.004), indicating increased pulmonary vascular load. Lastly, the TAPSE/PAPs ratio was significantly lower in patients with reduced vitamin D levels (0.65 ± 0.13 vs. 0.72 ± 0.14; p = 0.021), consistent with impaired right ventricular-pulmonary coupling.

**Fig. 1:**
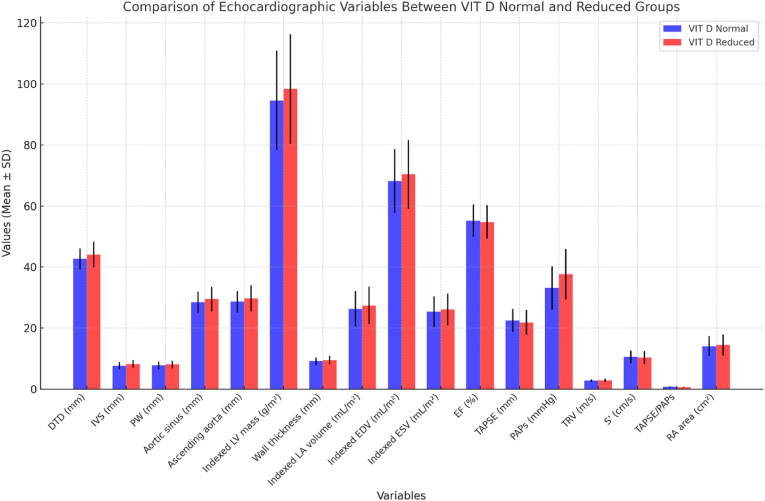
Left-ventricular structural echocardiographic variables in patients with normal vitamin D (≥30 ng/mL; n = 50) versus vitamin D insufficiency (<30 ng/mL; n = 110). Bars depict mean ± SD. Significant between-group differences were observed for LV end-diastolic diameter (LVEDD/DTD) and interventricular septal thickness (IVS), both higher in the insufficiency group (**p = 0.037** and **p = 0.028**, respectively). Posterior wall thickness and indexed LV mass showed no between-group differences (all **p ≥ 0.05**). Group comparisons used Student's *t*-test or Mann–Whitney *U* test, as appropriate; significance was set at **p < 0.05**.

These echocardiographic findings collectively suggest a pattern of combined subclinical left and right ventricular dysfunction in patients with hypovitaminosis D, as summarized in Fig. 1. Table 2.

The analysis of binary echocardiographic variables in patients with systemic sclerosis revealed no statistically significant differences between the groups with reduced and non-reduced vitamin D levels (*p*-value >0.05). Table 3.

**Fig. 2:**
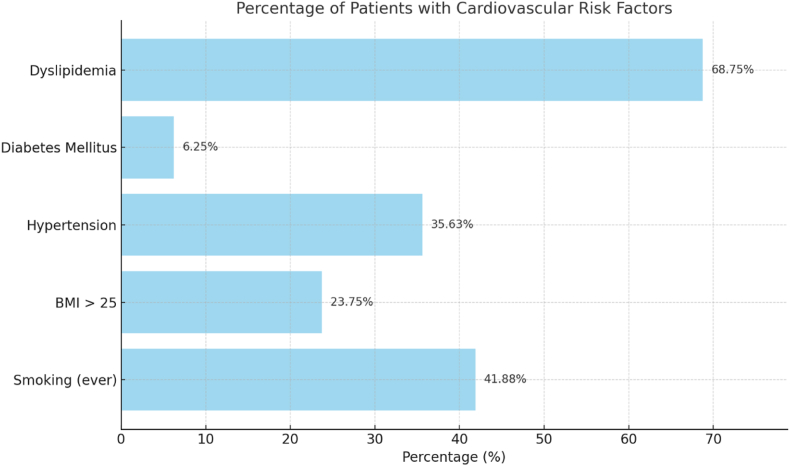
Prevalence of cardiovascular risk factors in the study cohort (n = 160). Bars depict the proportion of patients with each risk factor: smoking **41.9 % (n = 67)**, BMI >25 kg/m^2^**23.8 % (n = 38)**, hypertension **35.6 % (n = 57)**, diabetes mellitus **6.3 % (n = 10)**, and dyslipidemia **68.8 % (n = 110).**

Smoking (current or past) was present in 41.88 % of patients, a BMI >25, indicative of overweight or obesity, was observed in 23.75 %, arterial hypertension was detected in 35.63 %, diabetes mellitus was present in 6.25 %, and dyslipidemia was observed in 68.75 % of patients, as summarized in Fig. 2.

We analyzed the correlation between reduced vitamin D levels and cardiovascular risk factors using Pearson's correlation coefficient. Although the examined variables were binary categorical, they were treated as numerical data to estimate the strength and direction of the linear association. Pearson's coefficient was chosen because it allows for a standardized and easily interpretable quantification of the relationship: values close to zero indicate a weak or absent correlation, whereas higher values reflect a stronger association. This approach also facilitates comparison with similar analyses in the existing literature.

In addition to this analysis, the Fisher's exact test was used to assess the association between two binary categorical variables. This test provides a robust measure of statistical significance, independent of data distribution or sample size, ensuring greater reliability in the results. Table 4.

The Pearson correlation coefficient between “Reduced vitamin D″ and “BMI >25” is 0.234, indicating a weak positive correlation between low vitamin D levels and overweight/obesity. The odds ratio (OR) of 4.73 suggests that patients with reduced vitamin D levels have a significantly higher likelihood of being overweight/obese compared to those with normal vitamin D levels. The p-value of 0.002 confirms the association between reduced vitamin D levels and overweight/obesity.

The Pearson correlation coefficient between “Reduced vitamin D″ and “Smoking (never/former/current)” is 0.165, indicating a very weak positive correlation between low vitamin D levels and smoking (current or past). The odds ratio was not calculated because the p-value of 0.06 does not confirm a significant association between reduced vitamin D levels and smoking. This suggests that the observed result could be due to chance.

The Pearson correlation coefficient between “Reduced vitamin D″ and “Diabetes Mellitus (DM)” is 0.11, indicating a very weak positive correlation between low vitamin D levels and diabetes. The odds ratio was not calculated because the p-value of 0.28 does not support a significant association between reduced vitamin D levels and diabetes. Therefore, it is not possible to conclude that patients with low vitamin D levels are more likely to have diabetes than those with normal levels.

The Pearson correlation coefficient between “Reduced vitamin D″ and “Hypertension” is 0.17, indicating a weak positive correlation between low vitamin D levels and hypertension. The odds ratio of 2.31 suggests that patients with reduced vitamin D levels have a significantly higher likelihood of having hypertension compared to those with normal vitamin D levels. The p-value of 0.032 confirms the association between reduced vitamin D levels and hypertension.

The Pearson correlation coefficient between “Reduced vitamin D” and “Dyslipidemia” is 0.198, indicating a weak positive correlation between low vitamin D levels and dyslipidemia. The odds ratio of 2.45 suggests that patients with reduced vitamin D levels have a significantly higher likelihood of having dyslipidemia compared to those with normal vitamin D levels. The p-value of 0.015 confirms the association between reduced vitamin D levels and dyslipidemia.

The Pearson correlation coefficient between “Reduced vitamin D” and “eGFR <60 mL/min/1.73 m^2^” is 0.09, indicating a very weak positive correlation. The odds ratio was not calculated because the p-value of 0.21 does not indicate a statistically significant association.

The Pearson correlation coefficient between “Reduced vitamin D″ and “Antihypertensive therapy” is 0.16, indicating a weak positive correlation. The odds ratio of 2.12 confirms that patients with reduced vitamin D levels are more likely to be on antihypertensive therapy compared to those with normal vitamin D levels (p = 0.041).

The Pearson correlation coefficient between “Reduced vitamin D″ and “Lipid-lowering therapy” is 0.14, indicating a weak positive correlation. The odds ratio of 1.88 suggests that patients with reduced vitamin D levels have a higher likelihood of receiving lipid-lowering therapy compared to those with normal vitamin D levels (p = 0.045).

The Pearson correlation coefficient between “Reduced vitamin D″ and “Winter blood sampling” is 0.12, indicating a very weak positive correlation. The odds ratio was not calculated because the p-value of 0.08 does not support a significant association.

These findings underscore the significant relationship between vitamin D insufficiency and specific cardiovascular risk factors and treatments—particularly overweight/obesity, hypertension, dyslipidemia, and the use of antihypertensive or lipid-lowering drugs—reinforcing the hypothesis of vitamin D's role in cardiovascular risk modulation in systemic sclerosis.

## 4 Discussion

The primary findings of this study demonstrate that among a cohort of 160 patients with Systemic Sclerosis (SSc), a significant proportion (68.9 %) exhibited vitamin D insufficiency. Echocardiographic assessment revealed notable alterations indicative of cardiac impairment, particularly affecting the right ventricular function, as evidenced by elevated systolic pulmonary arterial pressure (PAPs: 37.68 ± 7.56 mmHg vs. 33.12 ± 6.17 mmHg; p = 0.004) and reduced TAPSE/PAPs ratio (0.65 ± 0.13 vs. 0.72 ± 0.14; p = 0.021) compared to patients with normal vitamin D levels. Additionally, structural modifications of the left ventricle were observed, including increased interventricular septal thickness (8.17 ± 1.28 mm vs. 7.69 ± 1.18 mm; p = 0.028) and larger left ventricular end-diastolic diameter (44.06 ± 4.28 mm vs. 42.67 ± 3.44 mm; p = 0.037). These findings underscore the multifaceted impact of vitamin D deficiency, which appears to influence both the structural and functional components of the cardiovascular system.

These echocardiographic changes are consistent with previous reports in the literature. Vitamin D has been shown to play a protective role in right ventricular function through modulation of inflammatory pathways and enhancement of endothelial function [33,34].

The observed increase in interventricular septal thickness may reflect early myocardial hypertrophy or fibrosis, possibly driven by chronic low-grade inflammation and impaired calcium homeostasis secondary to vitamin D deficiency. Similarly, a larger left ventricular end-diastolic diameter could suggest incipient ventricular remodeling associated with altered myocardial compliance. Experimental studies have demonstrated that vitamin D receptor (VDR) signaling within cardiomyocytes is essential for maintaining myocardial architecture and contractility. Vitamin D influences calcium handling and myocardial relaxation through both genomic and non-genomic pathways, and its deficiency could thus result in impaired ventricular mechanics and progressive dilation [8,9].

In the right heart, the elevated PAPs observed in vitamin D-deficient patients likely reflects increased pulmonary vascular resistance resulting from endothelial dysfunction and heightened oxidative stress, mechanisms that vitamin D typically mitigates. The reduced TAPSE/PAPs ratio further supports the hypothesis of impaired right ventricular-pulmonary arterial coupling in the setting of increased afterload. This maladaptive response may be exacerbated by vitamin D deficiency through pathways involving inflammation, fibrosis, and altered right ventricular mechanics [7,33,35].

It is important to acknowledge that the echocardiographic alterations observed in our cohort—such as increased systolic pulmonary arterial pressure and reduced TAPSE/PAPs ratio—may not be solely attributable to vitamin D insufficiency. Systemic sclerosis is characterized by intrinsic microvascular and endothelial dysfunction that can independently induce pulmonary vascular remodeling and right ventricular–pulmonary arterial uncoupling. SSc-associated vasculopathy often leads to intimal proliferation, medial hypertrophy, and luminal narrowing of small pulmonary arteries, culminating in increased pulmonary vascular resistance and eventual pulmonary hypertension. Such pathophysiological changes have been described as early and central features of the disease process [36,37].

Moreover, pulmonary arterial hypertension (PAH) develops in approximately 8–12 % of SSc patients and represents a major cause of morbidity and mortality, often occurring alongside endothelial injury and microangiopathic changes detectable via nailfold videocapillaroscopy [38].

Additionally, interstitial lung disease and chronic hypoxia—frequent complications in SSc—may further elevate pulmonary pressures and exacerbate right ventricular load, independently contributing to the echocardiographic findings observed. While the prevalence of elevated PAPs and reduced TAPSE/PAPs ratio was higher in the vitamin D–deficient subgroup, this association likely reflects a complex interplay between vitamin D metabolism and an underlying vascular pathogenetic process, rather than a direct causal relationship [39].

Taken together, these findings indicate that vitamin D insufficiency may contribute not only to pulmonary hypertension and right ventricular dysfunction but also to early alterations in left ventricular structure and function. This observation aligns with prior studies associating vitamin D deficiency in SSc with reduced bone mineral density and increased cardiovascular risk. Moreover, this study adds to the existing literature by explicitly documenting early structural changes in the left ventricle, thereby broadening the spectrum of cardiovascular manifestations associated with vitamin D deficiency in this patient population [35,[40], [41], [42]].

Systemic sclerosis itself comprises two major clinical subsets—limited cutaneous (lcSSc) and diffuse cutaneous (dcSSc)—each characterized by distinct patterns of organ involvement and prognosis. Notably, dcSSc is more frequently associated with profound vitamin D deficiency, likely due to more extensive gastrointestinal involvement, malabsorption, and severe skin fibrosis limiting cutaneous vitamin D synthesis. Furthermore, chronic systemic inflammation in dcSSc may increase vitamin D utilization and degradation [21,22,43,44].

In light of these data, careful monitoring of vitamin D status in SSc patients should be considered an essential component of cardiovascular risk assessment. Our findings support the potential utility of vitamin D supplementation as a means to improve not only skeletal health but also cardiovascular outcomes in this high-risk population.

Regarding the relationship between reduced vitamin D levels and cardiovascular risk factors, our analysis revealed weak yet statistically significant associations with overweight/obesity, hypertension, and dyslipidemia. The most pronounced association was observed between vitamin D insufficiency and overweight/obesity (odds ratio = 4.73; p = 0.002), in line with existing evidence that vitamin D becomes sequestered in adipose tissue, reducing its bioavailability, and that obesity is associated with a chronic inflammatory state [45]. Obesity has been shown to contribute to the decreased bioavailability of vitamin D, negatively affecting bone metabolism and the endocrine system [5].

Hypertension (odds ratio = 2.31; p = 0.032) was also significantly associated with low vitamin D levels. This is consistent with mechanistic studies demonstrating that vitamin D negatively regulates the renin-angiotensin-aldosterone system (RAAS), thereby reducing vasoconstriction and arterial pressure. Vitamin D deficiency has also been linked to endothelial dysfunction and arterial stiffness, key contributors to elevated blood pressure and cardiovascular morbidity [4,6].

Lastly, the association between vitamin D insufficiency and dyslipidemia (odds ratio = 2.45; p = 0.015) underscores a potential role for vitamin D in lipid metabolism and oxidative stress regulation. Several studies suggest that vitamin D may enhance endothelial function and reduce pro-atherogenic lipid profiles, thus mitigating atherosclerotic cardiovascular risk [7].

Similarly, the association between low vitamin D levels and the use of antihypertensive therapy (odds ratio = 2.12; p = 0.041) and lipid-lowering therapy (odds ratio = 1.88; p = 0.045) suggests that patients with vitamin D insufficiency may have a greater overall imbalance in cardiovascular status. Although the correlation with winter blood sampling and eGFR <60 mL/min/1.73 m^2^ did not reach statistical significance, both trends are consistent with the well-known biological influences on vitamin D status [46,47].

## 5 Limitations of the study

This study presents several limitations that should be acknowledged when interpreting the results.

First, although we collected additional information on renal function, residential latitude, season of blood sampling, and the use of antihypertensive and lipid-lowering therapies, other potential confounding factors were not available. These include dietary intake of calcium and phosphorus, direct quantification of ultraviolet light exposure, and detailed measures of central adiposity (e.g., waist circumference or waist-to-hip ratio). If available, inclusion of waist circumference or a proxy for ultraviolet exposure, such as self-reported average time spent outdoors, could further refine the analysis. All these factors are known to influence vitamin D status and could have partially affected the associations observed.

Second, the retrospective observational design does not allow for establishing a causal relationship between vitamin D insufficiency and the echocardiographic alterations or cardiovascular risk factors described.

Third, vitamin D deficiency and insufficiency are common in the general population, especially in elderly individuals, and the absence of a matched healthy control group prevents us from definitively attributing the higher prevalence observed to systemic sclerosis itself.

Fourth, certain clinical parameters that could further refine cardiovascular risk assessment—such as 24-h blood pressure monitoring, arterial stiffness indices, or more advanced cardiac imaging techniques—were not systematically collected.

Fifth, although echocardiographic assessments were performed by experienced cardiologists and in accordance with international guidelines, inter-observer variability was not formally tested, which may slightly limit reproducibility.

Finally, the sample size, although sufficient for detecting the main associations, may be underpowered for identifying subtler relationships, particularly in subgroup analyses (e.g., by SSc subtype or sex). Prospective, multicenter studies with larger and more diverse cohorts, standardized measurement of potential confounders, and inclusion of matched controls are warranted to confirm and expand these findings.

## 6 Conclusions

This study highlighted a high prevalence of vitamin D insufficiency in patients with systemic sclerosis and identified a significant association between low vitamin D levels and echocardiographic alterations indicative of early cardiac involvement. Specifically, patients with reduced vitamin D levels exhibited higher systolic pulmonary arterial pressure (PAPs) and a lower TAPSE/PAPs ratio, suggesting a potential negative impact on right ventricular function. Additionally, vitamin D insufficiency was correlated with increased interventricular septal thickness and larger left ventricular end-diastolic diameter, indicating early structural changes in cardiac function.

The analysis of cardiovascular risk factors revealed a significant association between vitamin D insufficiency and hypertension, dyslipidemia, and overweight/obesity, supporting the role of vitamin D in modulating metabolic and vascular processes. However, no statistically significant correlation was found with diabetes mellitus or smoking habits.

Our findings strengthen the hypothesis that vitamin D may play a protective role in the cardiovascular system, influencing inflammation, oxidative stress, and endothelial function. While epidemiological studies have documented an association between vitamin D insufficiency and cardiovascular risk, the effectiveness of vitamin D supplementation in improving cardiovascular outcomes remains a subject of debate, with conflicting evidence from randomized clinical trials.

However, given the limited sample size and the retrospective observational nature of our study, these results should be interpreted with caution. Multiple confounding factors cannot be ruled out, and our findings alone cannot establish a clear cause-effect relationship. Larger prospective studies are necessary to confirm these observations and to assess whether correcting vitamin D insufficiency can effectively improve cardiac parameters and reduce cardiovascular risk in systemic sclerosis patients.

## CRediT authorship contribution statement

**Gianluca Pagnoni:** Writing – review & editing, Writing – original draft, Visualization, Validation, Supervision, Software, Resources, Project administration, Methodology, Investigation, Funding acquisition, Formal analysis, Data curation, Conceptualization. **Dilia Giuggioli:** Validation, Supervision, Conceptualization. **Marco de Pinto:** Supervision, Project administration, Formal analysis, Data curation. **Arianna Maini:** Software, Project administration, Methodology, Data curation. **Elisa Battigaglia:** Project administration, Formal analysis, Data curation. **Pierluca Macripò:** Software, Formal analysis, Data curation. **Amelia Spinella:** Methodology, Investigation, Formal analysis, Data curation. **Giulia Olivetti:** Visualization, Validation, Software, Conceptualization. **Antonio Manenti:** Visualization, Validation, Supervision, Methodology, Investigation, Formal analysis. **Marcello Pinti:** Writing – original draft, Visualization, Validation, Supervision, Resources, Investigation, Funding acquisition. **Giuseppe Boriani:** Validation, Supervision. **Daniela Aschieri:** Visualization, Validation, Supervision, Investigation. **Anna Vittoria Mattioli:** Writing – original draft, Visualization, Validation, Supervision, Resources, Methodology, Conceptualization. **Francesco Fedele:** Visualization, Validation, Supervision, Resources, Funding acquisition. **Francesca Coppi:** Writing – review & editing, Writing – original draft, Visualization, Validation, Supervision, Software, Resources, Project administration, Methodology, Investigation, Funding acquisition, Formal analysis, Data curation, Conceptualization.

## Data availability

De-identified data are available from the corresponding author upon reasonable request.

## Funding

The research leading to these results has received funding from.

• The European Union - NextGenerationEU through the Italian Ministry of University and Research under PNRR - M4C2–I1.3 Project PE_00000019 "HEAL ITALIA" (D.G., A.V.M., M.P. and F.C.).
• National Institute for Cardiovascular Research (INRC), Via Irnerio 48, 40126, Bologna (Italy)

## Declaration of competing interest

The authors declare no conflicts of interest related to this work.
